# Risk Profiles and Antithrombotic Treatment of Patients Newly Diagnosed with Atrial Fibrillation at Risk of Stroke: Perspectives from the International, Observational, Prospective GARFIELD Registry

**DOI:** 10.1371/journal.pone.0063479

**Published:** 2013-05-21

**Authors:** Ajay K. Kakkar, Iris Mueller, Jean-Pierre Bassand, David A. Fitzmaurice, Samuel Z. Goldhaber, Shinya Goto, Sylvia Haas, Werner Hacke, Gregory Y. H. Lip, Lorenzo G. Mantovani, Alexander G. G. Turpie, Martin van Eickels, Frank Misselwitz, Sophie Rushton-Smith, Gloria Kayani, Peter Wilkinson, Freek W. A. Verheugt, for the GARFIELD Registry Investigators

**Affiliations:** 1 Thrombosis Research Institute, London, United Kingdom; 2 University College London, London, United Kingdom; 3 Department of Cardiology, University Hospital Jean-Minjoz, Besançon, France; 4 Primary Care Clinical Sciences, The University of Birmingham, Birmingham, United Kingdom; 5 Department of Medicine, Harvard Medical School, Boston, Massachusetts, and Department of Medicine, Brigham and Women’s Hospital, Boston, Massachusetts, United States of America; 6 Department of Medicine, Tokai University, Kanagawa, Japan; 7 Department of Medicine, Technical University of Munich, Munich, Germany; 8 Department of Neurology, University Hospital of Heidelberg, Heidelberg, Germany; 9 University of Birmingham Centre for Cardiovascular Sciences, City Hospital, Birmingham, United Kingdom; 10 Dipartimento di Medicina Clinica e Chirurgia, Università degli Studi di Napoli Federico II, Napoli, Italy; 11 Department of Medicine, McMaster University, Hamilton, Ontario, Canada; 12 Bayer HealthCare Pharmaceuticals, Berlin, Germany; 13 Wilkinson Associates, Radnage, Bucks, United Kingdom; 14 Department of Cardiology, Onze Lieve Vrouwe Gasthuis (OLVG), Amsterdam, The Netherlands; Universidad Peruana de Ciencias Aplicadas (UPC), Peru

## Abstract

**Background:**

Limited data are available on the characteristics, clinical management, and outcomes of patients with atrial fibrillation at risk of stroke, from a worldwide perspective. The aim of this study was to describe the baseline characteristics and initial therapeutic management of patients with non-valvular atrial fibrillation across the spectrum of sites at which these patients are treated.

**Methods and Findings:**

The Global Anticoagulant Registry in the FIELD (GARFIELD) is an observational study of patients newly diagnosed with non-valvular atrial fibrillation. Enrollment into Cohort 1 (of 5) took place between December 2009 and October 2011 at 540 sites in 19 countries in Europe, Asia-Pacific, Central/South America, and Canada. Investigator sites are representative of the distribution of atrial fibrillation care settings in each country. Cohort 1 comprised 10,614 adults (≥18 years) diagnosed with non-valvular atrial fibrillation within the previous 6 weeks, with ≥1 investigator-defined stroke risk factor (not limited to those in existing risk-stratification schemes), and regardless of therapy. Data collected at baseline included demographics, medical history, care setting, nature of atrial fibrillation, and treatments initiated at diagnosis. The mean (SD) age of the population was 70.2 (11.2) years; 43.2% were women. Mean±SD CHADS_2_ score was 1.9±1.2, and 57.2% had a score ≥2. Mean CHA_2_DS_2_-VASc score was 3.2±1.6, and 8,957 (84.4%) had a score ≥2. Overall, 38.0% of patients with a CHADS_2_ score ≥2 did not receive anticoagulant therapy, whereas 42.5% of those at low risk (score 0) received anticoagulant therapy.

**Conclusions:**

These contemporary observational worldwide data on non-valvular atrial fibrillation, collected at the end of the vitamin K antagonist-only era, indicate that these drugs are frequently not being used according to stroke risk scores and guidelines, with overuse in patients at low risk and underuse in those at high risk of stroke.

**Trial Registration:**

ClinicalTrials.gov TRI08888

## Introduction

Atrial fibrillation (AF) is the most common heart rhythm disorder, with approximately one-quarter of individuals over 40 years of age developing this arrhythmia [1]. The risk of stroke – including ischemic stroke, hemorrhagic stroke and cerebral bleeds – increases fivefold among patients with AF [2]. AF is also associated with a twofold excess risk of cardiovascular death and stroke within 1 year of observation [3].

Vitamin K antagonists (VKAs) have served as the cornerstone of stroke prevention in AF for several decades. Comprehensive evidence-based management guidelines [4], [5], [6], [7] advocating the use of risk scores to identify patients most (or least) at risk of thrombotic or bleeding events are widely available. VKAs have a number of drawbacks, however, including a narrow therapeutic window, multiple food and drug interactions [8], and substantial inter-patient variability due to genetic or other factors, making their long-term use in clinical practice a challenge [9]. Physicians remain reluctant to prescribe anticoagulant prophylaxis in a large proportion of the population at risk for stroke, in part due to the limitations of VKAs, misperception of thrombotic risk [10], and concern about bleeding complications, especially among the elderly [11].

International observational studies have provided insights into the characteristics, risk profiles, management, and clinical outcomes of patients with various cardiovascular diseases [XPATH ERROR: unknown variable "start2".], [13]. Less is known about individuals newly diagnosed with AF and perceived to be at risk of stroke by their physicians, and few data are available that reflect the broad range of healthcare settings for AF from a worldwide perspective.

The Global Anticoagulant Registry in the FIELD (GARFIELD) was initiated to describe everyday antithrombotic treatment patterns in patients newly diagnosed with non-valvular AF and one or more additional investigator-defined stroke risk factor across the spectrum of care settings at which these patients are treated, and to understand the burden of thromboembolic and bleeding complications in this population. This article presents the baseline characteristics and initial management of the first of five cohorts of over 10,000 patients enrolled in the GARFIELD Registry.

## Methods

### Ethics Statement

Independent ethics committee and hospital-based institutional review board approvals were obtained, as necessary, for the registry protocol. (See Ethics List S1) The registry is being conducted in accordance with the principles of the Declaration of Helsinki, local regulatory requirements, and the International Conference on Harmonisation–Good Pharmacoepidemiological and Clinical Practice guidelines. All patients provided written informed consent to participate.

### Trial Design and Participants

The GARFIELD Registry is an ongoing, observational, multicenter, worldwide study of adults (≥18 years) with non-valvular AF diagnosed according to standard local procedures within the past 6 weeks (electrocardiogram confirmation was not mandated) and ≥1 additional factor judged by the clinician to increase the patient’s risk of stroke; such factors were not prespecified in the protocol, nor were they limited to the factors in risk-stratification schemes such as CHADS_2_ (cardiac failure, hypertension, age, diabetes, stroke [doubled]) [14] or CHA_2_DS_2_-VASc (cardiac failure, hypertension, age ≥75 [doubled], diabetes, stroke [doubled]-vascular disease, age 65–74 and sex category [female]) [15].

Enrollment will take place in five independent, sequential cohorts [16]. Patient enrollment into Cohort 1 took place between 21 December 2009 and 26 October 2011. In parallel with prospectively enrolled patients, a validation group (part retrospective and part prospective) was enrolled, comprising patients with established AF (i.e., AF first diagnosed ≥6 months and ≤24 months before enrollment) and ≥1 additional risk factor for stroke, regardless of therapy; in these patients, data were collected retrospectively to the time of first AF diagnosis, and prospectively up to 2 years after diagnosis. The rationale for the inclusion of the retrospective cohort was to evaluate, by comparing retrospective with prospective data, whether initiation of the GARFIELD Registry influenced AF management patterns on a site level; if the effect was zero or minimal, the data from both cohorts would to be combined.

Patients for whom follow-up up to 2 years was unlikely and those with a transient reversible cause of AF were excluded. Data were collected using an electronic case report form (eCRF) [16].

Investigator sites are representative of the distribution of AF treating care settings in each country. Sufficient sites were identified from the spectrum of care settings (office-based practice, hospital departments [neurology, cardiology, geriatrics, internal medicine, emergency], anticoagulant clinics, and general or family practice) to ensure proportional representation in all countries, and the lists and ratios were validated by national coordinators [16]. Sites were selected randomly and recruited following a qualification call. Before site initiation, investigators were required to complete a training program that provided guidance on patient screening, enrollment, and follow-up. Patients were enrolled consecutively, as stipulated in the protocol.

### Procedures

Data collected at baseline included patient and clinical characteristics at diagnosis, medical history (including cardiovascular and bleeding history), care setting at diagnosis, type of AF, date and method of diagnosis, symptoms, antithrombotic treatment at diagnosis (VKAs, factor Xa inhibitors, thrombin inhibitors, and heparins), and reasons for not providing VKAs (when applicable). Ethnicity was classified by the investigator, in agreement with the patient, to investigate ethnic differences in the prevalence of AF [17].

Heart failure, hypertension (blood pressure >140/90 mmHg or treated hypertension), age ≥75 years, diabetes mellitus, and prior stroke or transient ischemic attack were used to calculate, retrospectively, stroke risk according to the CHADS_2_ risk index [14]. Additionally, left ventricular ejection fraction <40%, prior thromboembolism, vascular disease (acute coronary syndrome, peripheral artery disease), age 65–74 years, and female gender were used to determine stroke risk using the CHA_2_DS_2_-VASc score [15].

Registry data were captured in electronic CRFs (designed by Dendrite Clinical Systems Ltd, Henley-on-Thames, UK, who are also responsible for ongoing database programme management). Data collection and entry are managed by Quintiles (Durham, NC, USA), who oversee all operational aspects of the programme, apart from in the UK where these aspects are undertaken by The University of Birmingham Department of Primary Care Clinical Sciences. Submitted data are examined by the coordinating center (Thrombosis Research Institute, London) to ascertain their completeness and accuracy, and data queries are sent to participating sites. Data are extracted for each analysis and analyzed by an independent statistician (PW). Confidentiality and anonymity of all patients enrolled into this registry was maintained at all times.

### Statistical Analysis

Continuous variables are expressed as mean±standard deviation (SD). Categorical variables are expressed as frequencies and percentages. Differences between cohorts were tested for statistical significance using the Chi-squared test for categorical variables and the unpaired *t*-test for continuous variables. Statistical analysis was performed using SAS® software version 9.1.3 (SAS Institute Inc., Cary, NC, USA).

## Results

**Figure 1:**
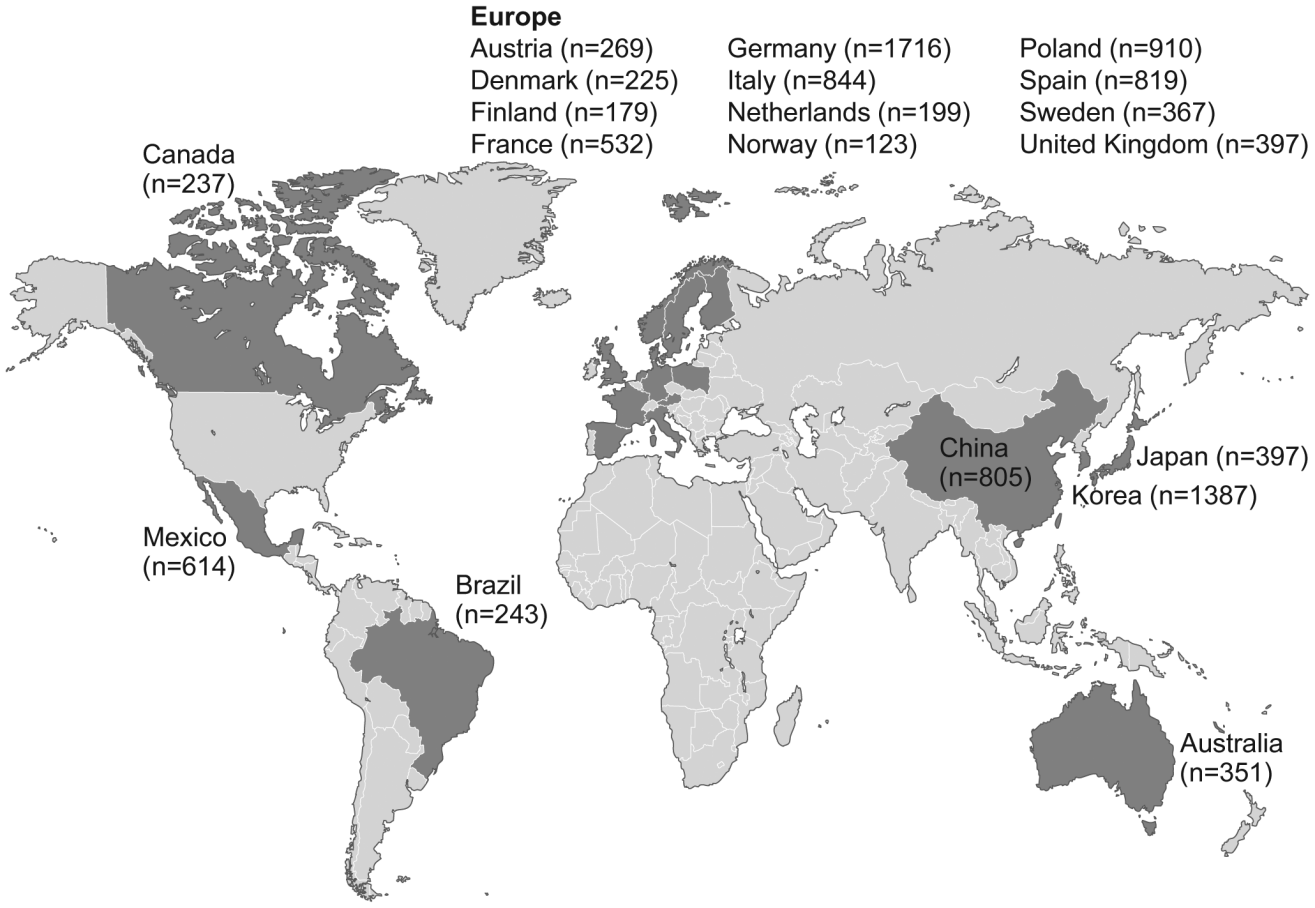
Number of patients enrolled per country in Cohort 1 (n = 10,614).

A total of 10,614 patients were enrolled into Cohort 1 at 540 sites in 19 countries in Asia-Pacific (n = 2,940, 27.7%; Australia, China, Korea, Japan), Canada (n = 237, 2.2%), Europe (n = 6,580, 62.0%; Austria, Denmark, Finland, France, Germany, Italy, Netherlands, Norway, Poland, Spain, Sweden, UK), and Central/South America (n = 857, 8.1%; Brazil, Mexico) (Figure 1). More than half of the patients (n = 6,262, 59.0%) were enrolled by cardiologists, 20.8% (n = 2,208) by internal medicine specialists, 17.7% (n = 1,880) by primary care/general practice physicians, 2.1% (n = 218) by neurologists, and 0.4% by geriatricians (n = 40) (data for six patients unknown). Each site recruited 20 consecutive patients on average. Baseline data were locked in 99.9% of the patients.

Overall, 29.7% of patients had new or unclassified AF, 27.5% had paroxysmal AF, 17.9% had persistent AF, and 24.9% had permanent AF. White patients represented the largest percentage of the population (62.2%), followed by Asians (7.6% Chinese and 17.0% other Asian ethnicities); the remaining patients were White–Hispanic/Latino (8.2%), mixed/other (1.2%), Afro-Caribbean (0.3%), or of unknown race (3.5%).

Of the overall cohort of 10,614 patients, 5,089 (47.9%) were enrolled retrospectively and 5,525 (52.1%) prospectively (see Figure S1 for year of diagnosis). No statistical or clinical concerns were apparent regarding any of the differences in baseline characteristics of the retrospectively and prospectively enrolled patients that would preclude combining the data from these two groups (Table S1, Figure S2), hence combined results are reported.

**Figure 2:**
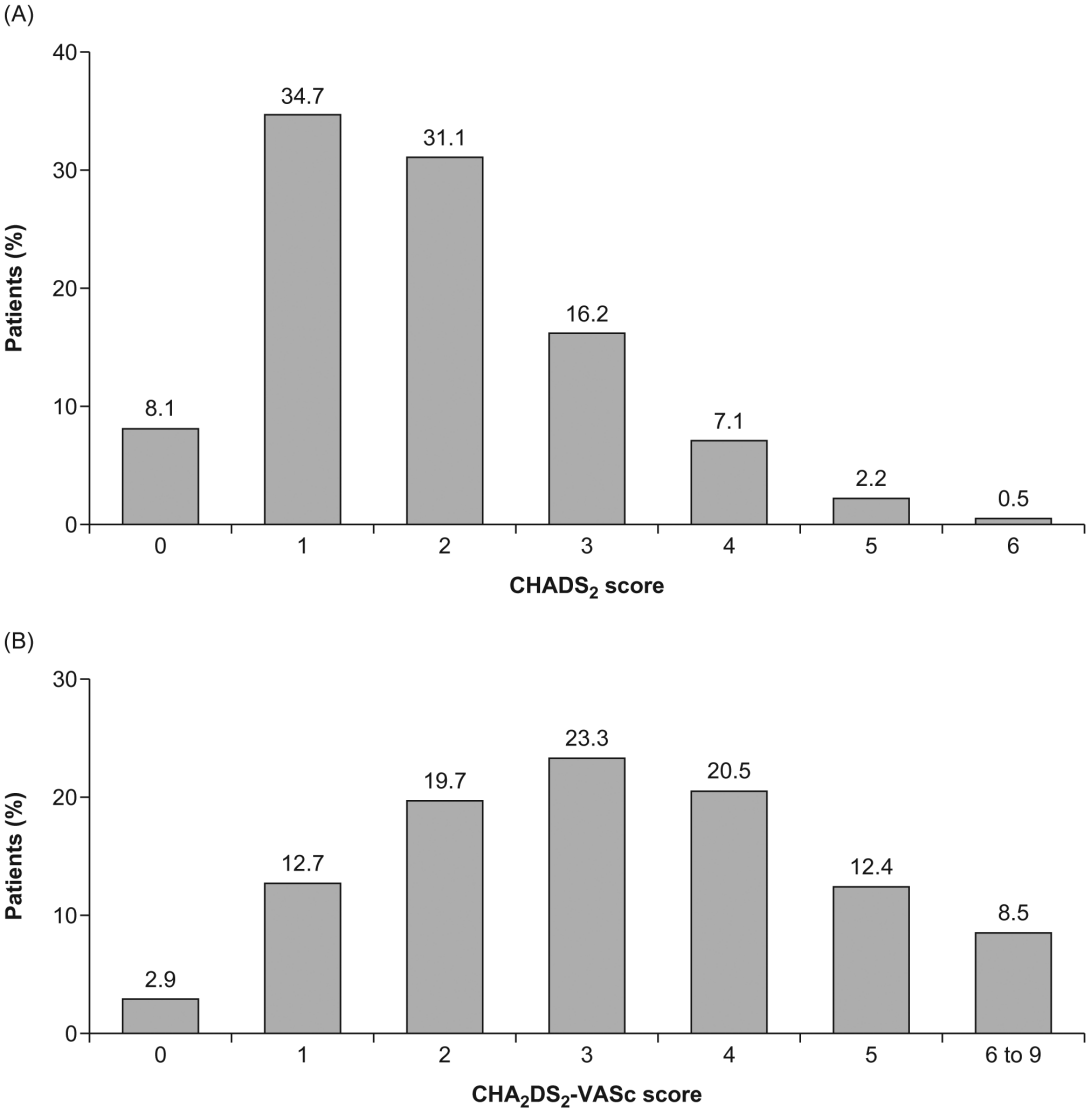
Distribution of CHADS_2_ and CHA_2_DS_2_-VASc scores (n = 10,607) (scores not available for 7 patients).

Baseline characteristics are given in Table 1. The mean±SD age was 70.2±11.2 years and 69.5% of patients were >65 years; 43.2% were women. More than three-quarters (77.8%) of the population had hypertension, 22.0% had diabetes mellitus, 21.0% had congestive heart failure, and 9.7% of patients had a history of stroke. Over one-third (35.2%) of patients were current or previous smokers. The mean±SD CHADS_2_ score was 1.9±1.2, and 57.2% (6,062/10,607) of patients had a score ≥2, indicating a moderate to high risk of stroke and guideline qualification for oral anticoagulant treatment. Mean±SD CHA_2_DS_2_-VASc score was 3.2±1.6, and 84.4% (8,957/10,607) had a score ≥2. The distributions of risk scores for stroke are shown in Figure 2.

**Table 1:** Patient baseline characteristics: Cohort 1 of the GARFIELD Registry.

Variable	All patients (n = 10,614)
Age, mean (SD), years	70.2±11.2
Age group, *n* (%)	
>65 years	7,374 (69.5)
≥75 years	4,091 (38.5)
65–74 years	3,540 (33.4)
Women, *n* (%)	4,580 (43.2)
Body mass index,^a^ mean (SD), kg/m^2^	27.5±5.3
Smoking status (current/previous)^b^, *n* (%)	3,504 (35.2)
Pulse,^c^ mean (SD), bpm	86.6±25.1
Medical history, *n* (%)	
Acute coronary syndromes (myocardial infarction or unstable angina)	1,060 (10.0)
Congestive heart failure^d^	2,229 (21.0)
Coronary artery disease^d^	2,035 (19.2)
Hypercholesterolemia^d^	4,159 (39.2)
Hypertension^d^	8,249 (77.8)
Family history of cardiac diseasee,f	1,940 (18.3)
Diabetes mellitus^f^	2,330 (22.0)
Stroke^d^	1,026 (9.7)
Stroke or transient ischaemic attack^d^	1,528 (14.4)
Left ventricular ejection fraction <40%^g^	586 (9.5)
Chronic renal disease^h^	
Mild renal dysfunction (GFR 60–89 mL/min)	1,502 (19.6)
Moderate renal dysfunction (GFR 30–59 mL/min)	871 (11.4)
Severe renal dysfunction or renal failure (GFR <30 mL/min)	154 (2.0)
Peripheral artery disease^d^	743 (7.0)
Carotid occlusive disease^d^	368 (3.5)
Other thromboembolismd,i	150 (1.4)
Systemic embolism^d^	80 (0.8)
Pulmonary embolism or deep vein thrombosis^d^	304 (2.9)
Bleeding^d^	368 (3.5)
Heavy alcohol consumption^j^	215 (2.2)
Cirrhosis^d^	55 (0.5)

Abbreviation: GFR, glomerular filtration rate.

a Data not available for 1,611 patients.

b Data not available for 671 patients.

c Data not available for 1,372 patients.

d Data not available for 6 patients.

e First-degree relative with premature cardiac history (age <55 years [male], <65 years [female]).

f Data not available for 7 patients.

g Data not available for 4,448 patients.

h Data not available for 2,954 patients.

i For example, central venous thrombosis, retinal occlusion.

j Investigator defined; data not available for 1,048 patients.

**Figure 3:**
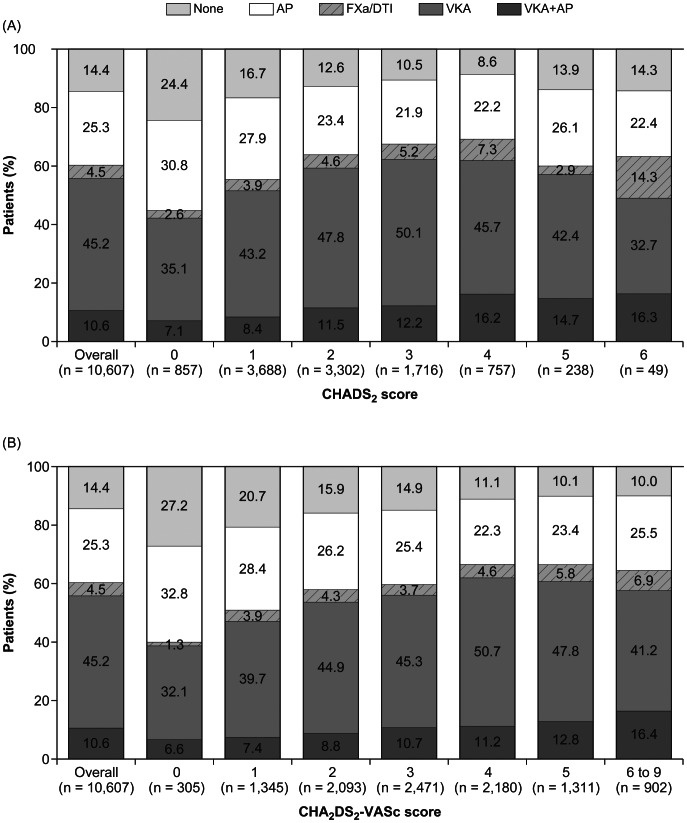
Use of antithrombotic therapies, overall and according to (A) CHADS_2_ score and (B) CHA_2_DS_2_VASc score (n = 10,607). AP indicates antiplatelet; FXa/DTI, activated coagulation factor X inhibitor/direct thrombin inhibitor (irrespective of AP use); VKA, vitamin K antagonist.

At diagnosis, 55.8% of patients overall were given a VKA for stroke prevention: 45.2% (n = 4,797) received a VKA alone and 10.6% (n = 1,128) received both a VKA and an antiplatelet drug (Figure 3). A minority of patients (4.5%, n = 475) received a novel oral factor Xa inhibitor or direct thrombin inhibitor. Just over one-quarter (25.3%, n = 2,681) of the patients received an antiplatelet drug alone and 14.4% (n = 1,533) received none of these antithrombotic drugs. Use of antithrombotic drugs at AF diagnosis and contraindications to anticoagulant therapy are detailed in Table 2. The most frequently given antiplatelet was aspirin. Use of all antithrombotic drugs was higher in patients with a CHADS_2_ score of 2–6 versus those with a score of 0 or 1. Contraindications to anticoagulant therapy were reported in 827 (7.8%) of patients.

**Table 2:** Use of antithrombotic drugs at diagnosis and contraindications to anticoagulant therapy, overall and according to CHADS_2_ scores of 0 or 1 and 2–6.

Drug	All patients (n = 10,614)	Patients with CHADS_2_score of 0 or 1 (n = 4,367)	Patients with CHADS_2_score of 2–6 (n = 6,240)
Antiplateleta,b, *n* (%)			
Aspirin	2,713 (26.5)	1166 (28.4)	1547 (25.2)
Thienopyridine	713 (7.0)	229 (5.6)	484 (7.9)
Glycoprotein IIb/IIIa inhibitor	16 (0.2)	6 (0.1)	10 (0.2)
Prostaglandin analogue	18 (0.2)	6 (0.1)	12 (0.2)
Other antiplatelet	102 (1.0)	30 (0.7)	72 (1.2)
Anticoagulant drugsa,c, *n* (%)			
Vitamin K antagonist	6,080 (58.2)	2218 (52.8)	3861 (61.9)
Heparin (unfractionated or low-molecular-weight)	410 (3.9)	151 (3.6)	259 (4.2)
Factor Xa inhibitor (oral or injectable)	312 (3.0)	102 (2.4)	210 (3.4)
Direct thrombin inhibitor (e.g., argatroban, dabigatran,bivalirudin, desirudin)	128 (1.2)	43 (1.0)	85 (1.4)
Heparinoid (e.g., danaparoid, sulodexide, dermatan sulfate)	89 (0.9)	35 (0.8)	54 (0.9)
Other anticoagulant (e.g., defibrotide, ramatroban,antithrombin III, protein C)	31 (0.3)	9 (0.2)	22 (0.4)
Contraindication to anticoagulant therapy, *n* (%)^a^			
Excessive bleeding risk	289 (2.7)	88 (2.0)	201 (3.2)
Frequent falls or mechanical risk	238 (2.2)	38 (0.9)	200 (3.2)
Risk of drug interaction	39 (0.4)	13 (0.3)	26 (0.4)
Allergy	11 (0.1)	2 (<0.1)	9 (0.1)
Other contraindication	249 (2.3)	76 (1.7)	174 (2.8)

a Categories are not mutually exclusive.

b Data not available for 360 patients.

c Data not available for 171 patients.

The use of anticoagulant therapy by CHADS_2_ risk score is shown in Figure 3A. VKA use (alone or with an antiplatelet) increased as risk level increased, up to a maximum of 63% at a CHADS_2_ score of 3 and 4, then decreased thereafter. Use of novel factor Xa inhibitors and direct thrombin inhibitors was low across all risk categories. Overall, 38.0% (2,302/6,062) of patients with a CHADS_2_ score ≥2 did not receive anticoagulant therapy; conversely, 42.5% (364/857) of those at low risk (score of 0) received anticoagulant therapy. Similar patterns were observed when risk was assessed according to the CHA_2_DS_2_-VASc score: 40.7% (3,645/8,957) of the patients with a score ≥2 did not receive anticoagulant therapy, and 38.7% (118/305) of patients with a score of 0 received anticoagulant therapy (Figure 3B). For both risk scores, use of antiplatelet therapy showed an initial decline with rising risk level.

The reasons for not providing VKA therapy to patients at moderate to high risk of stroke are given in Table 3. Almost half of the reasons for not providing VKA related to physician choice.

**Table 3:** Main reasons why vitamin K antagonists were not given in patients with a CHADS_2_ score ≥2.

Reason, n (%)	Patients with CHADS_2_≥2 (n = 2,302)
Alcohol misuse	11 (0.5)
Already taking antiplatelet drug for another medical condition	117 (5.1)
Patient refusal	165 (7.2)
Previous bleeding event	55 (2.4)
Taking medication contraindicated or cautioned for use with vitamin K antagonists	16 (0.7)
Other	239 (10.4)
Unknown	587 (25.5)
Physician’s choice	1,112 (48.3)
Bleeding risk	170 (7.4)
Concern over patient compliance	121 (5.3)
Guideline recommendation	32 (1.4)
Fall risk	150 (6.5)
Low risk of stroke	95 (4.1)
Other	544 (23.6)

## Discussion

This large, ongoing, international observational study of patients newly diagnosed with non-valvular AF and one or more additional risk factors for stroke provides a unique perspective of AF management at the end of the VKA-only era, transitioning into the period in which novel oral anticoagulants present an alternative treatment for stroke prevention. The data illustrate the high rate of comorbid conditions in this population, including hypertension, hypercholesterolemia, diabetes mellitus, heart failure, and coronary artery disease. A sizable proportion of the population had a history of stroke or transient ischemic attack. Nearly 6/10 patients presented with a CHADS_2_ score ≥2 and more than 8/10 with a CHA_2_DS_2_VASc score ≥2. These figures correlate with an annual adjusted stroke rate ranging from 3.4% for a CHADS_2_ score of 2 to 18.2% for a score of 6 [14], and from 2.2% for a CHA_2_DS_2_-VASc score of 2 to 15.2% for a score of 9 [15]. Despite this high level of thromboembolic risk, overall use of anticoagulant therapy was relatively low. A total of 40.7% of the patients with a CHA_2_DS_2_-VASc score ≥2 did not receive guideline-recommended anticoagulant prophylaxis [5], [6], [7], [18]. Conversely, 38.7% of the patients with a CHA_2_DS_2_-VASc score of 0 received anticoagulant therapy; these individuals are regarded as “truly” low-risk subjects, with a stroke/thromboembolism event rate of 0.84 (95% confidence interval [CI], 0.65–1.08) per 100 person-years in patients with AF, compared with 3.49 (95% CI, 3.31–3.68) per 100 person-years for AF patients with a CHADS_2_ score of 0 or 1 [19]. Our present study, in which risk stratification was done retrospectively through a review of data collected in the eCRFs, indicates that the identification in “real-world” practice of patients perceived to be at risk of stroke is often not based on evidence-based risk schemes and guidelines [6]. There appears to be overuse of anticoagulant therapy in patients at low risk of stroke or systemic embolism, and underuse in those at moderate to high risk. Physicians’ clinical judgment of stroke risk therefore appears to incorporate factors beyond those included in CHADS_2_ and CHA_2_DS_2_-VASc.

### Comparison of GARFIELD with Other Registries in Non-valvular AF

One of the largest multinational studies in this field is the Euro Heart Survey on Atrial Fibrillation, conducted between 2003 and 2004 in more than 5,000 ambulatory and hospitalized patients [20]. The Euro Heart Survey provided a snapshot of the management of AF across 35 European Society of Cardiology member countries and revealed discordance between guidelines and everyday clinical practice. Oral anticoagulation was prescribed in 67% of patients considered eligible for VKA treatment according to guidelines [4], but also in 49% of ineligible patients. The Euro Heart Survey did indicate an increase in use of oral anticoagulant therapy with increasing stroke risk [21], in contrast to the results of a large nationwide retrospective US medical claims database study involving over 171,000 patients with AF (51,907 of whom had newly diagnosed AF), which indicated a low use of warfarin across all risk categories (overall rate 42.6%; 49.5% in patients newly diagnosed with AF) [22]. Both studies were consistent, however, in reporting underuse of oral anticoagulation in patients at elevated risk and overuse in those at low risk. Our present data show no apparent improvement in adherence to evidence-based guidelines [5], [6], [18] for AF in recent years.

Despite mandating the presence of one or more additional stroke risk factors in an effort to exclude patients with lone AF, the GARFIELD population fares as relatively low risk compared with other observational cohorts, and is substantially lower risk than the populations included in randomized trials of novel oral anticoagulant drugs (Table 4) [23], [24], [25], [26]. “Additional risk factors” were investigator defined in order to identify patients that physicians themselves perceived – during the course of their usual practice – to be at risk of stroke. This approach contrasts with other studies that mandated the presence of one or more specific risk factors, such as prior stroke or transient ischemic attack, hypertension, and heart failure, resulting in much higher-risk populations than typically seen in everyday clinical practice. Further, other data sets have been based on patients identified in emergency departments or hospitalized for another condition, who may be at high risk of a poor outcome [27]. In the nationwide US Outcomes Registry for Better Treatment of Atrial Fibrillation (ORBIT-AF) [28], for example, the mean±SD CHADS_2_ score was 2.3±1.3 [28], the mean age was 76 years, and approximately 30% of patients had diabetes and 30% heart failure. In contrast, in GARFIELD, the mean CHADS_2_ score was 1.9±1.2, mean age was 70 years, and only 22% patients had diabetes and 21% had heart failure. The differences in these two study populations may be due the fact that GARFIELD enrolled patients newly diagnosed with AF whereas ORBIT-AF enrolled patients with prevalent or incident AF.

**Table 4:** Baseline characteristics: Randomized clinical trials versus the GARFIELD Registry.

	GARFIELD(Cohort 1)	RELY-AF [31] (dabigatran)			ROCKET AF [32] (rivaroxaban)		AVERROES [26] (apixaban)		ARISTOTLE (apixaban) [33]	
	(n = 10,614)	D 110(n = 6015)	D 150(n = 6076)	Warf(n = 6022)	Rivarox(n = 7131)	Warfarin(n = 7133)	Apixaban(n = 2808)	Aspirin(n = 2791)	Apixaban(n = 9120)	Warfarin(n = 9081)
Age in years	70±11	71±9	72±9	72±9	73 (65,78)	73 (65,78)	70±9	70±10	70 (63,76)	70 (63,76)
Women	43	36	37	37	40	40	41	42	36	35
BMI (kg/m^2^)	28±5	–	–	–	28 (25,32)	28 (25,32)	28±5	28±5	–	–
Age ≥75 years	39	–	–	–	–	–	–	–	31	31
Prior stroke/TIA	14	20	20	20	–	–	14	13	–	–
Diabetes	22	23	23	23	40	40	19	20	25	25
Prior myocardial infarction	10*	17	17	16	17	18	–	–	15	14
Hypertension	78	79	79	79	90	91	86	87	87	88
Heart failure	21	32	32	32	63	62	40	38	36†	35†
Classification of AF										
Paroxysmal	28	32	33	34	18	18	27	27	15	16
Persistent	18	32	31	32	81	81	21	21	85	84
Permanent	25	35	36	34	–	–	52	52		
Newly diagnosed or new onset	30	–	–	–	1.4	1.4	–	–	–	–
CHADS_2_ score	1.9±1.2	2.1±1.1	2.2±1.2	2.1±1.1	3.5±0.9	3.5±1.0	2.0±1.1	2.1±1.1	2.1±1.1	2.1±1.1

Data given as %, mean±SD or median (IQR).

* History of acute coronary syndrome.

† Heart failure or reduced left ventricular ejection fraction.

AF, atrial fibrillation; BMI, body mass index; CHADS_2_, Cardiac failure, Hypertension, Age, Diabetes, Stroke (Doubled), COPD, chronic obstructive pulmonary disease; D, dabigatran; SD, standard deviation; TIA, transient ischaemic attack.

RealiseAF was a large, international, contemporary, cross-sectional study conducted in 10,523 outpatients in 26 countries between 2009 and 2010 [29], with the aim of investigating the success of rhythm versus rate control, and the impact of control on patients’ clinical symptoms and quality of life. The patients in this registry were slightly younger than those in GARFIELD (67 vs. 70 years), and they had higher rates of heart failure (46% vs. 21%), coronary artery disease (32% vs. 19%) and hypercholesterolemia (46% vs. 39%); these differences in baseline characteristics likely reflect differences in the treatment settings. In RealiseAF, for example, all of the patients were enrolled by cardiologists or internists (hospital- and office-based), whereas in GARFIELD the aim was to reflect the spectrum of care settings in each country; consequently 80% of the patients were enrolled by cardiologists or internists, 18% were enrolled by primary care/general practice physicians, and 2.1% were enrolled by neurologists. Furthermore, 62% of the RealiseAF population was diagnosed with AF >12 months previously, whereas patients in GARFIELD were newly diagnosed, a finding supported by the higher rate of permanent AF in RealiseAF (46% vs. 25%).

Several other large registries in AF have been launched recently, including the international Global Registry on Long-Term Oral Antithrombotic Treatment in Patients with Atrial Fibrillation (GLORIA-AF), and the nationwide US Practice INNovation And Clinical Excellence (PINNACLE-AF) registry – part of the American College of Cardiology National Cardiovascular Data Registry. Physician participation in PINNACLE-AF is voluntary, and the study will report on outcomes in 121,000 patients with AF [30]. Like GARFIELD, PINNACLE-AF will rigorously assess current and evolving practice patterns; it will also help US providers evaluate and improve adherence to guidelines and performance measures through the provision of checklists of guideline-recommended care and provision of quarterly reports. These national US data will be complementary to GARFIELD, which will be conducted in up to 35 countries throughout the world.

### Future Insights from the GARFIELD Registry

The second GARFIELD cohort was initiated in October 2011, and an additional 11 countries (Argentina, Belgium, Chile, Czech Republic, Hungary, India, Russia, Singapore, South Africa, Thailand, Ukraine) joined the registry. Owing to its unique methodology, the GARFIELD Registry will continue to provide prospective and rigorous global and national data on “real-world” risk stratification, AF management, and clinical outcomes in patients newly diagnosed with AF and at risk of stroke.

### Study Limitations

As an observational study, GARFIELD is subject to certain limitations inherent in all such studies, such as the collection of non-randomized data and missing or incomplete information. Given the wide spectrum of care settings, the presence of missing or incomplete data is not surprising, particularly as site selection was random, encompassed the spectrum of care settings, and included sites with no clinical research experience. The study does, however, provide broader insights into the management of AF patients, with the inclusion of care environments not normally included in such studies. The data collected will provide a “snapshot” of anticoagulant use at the point of evaluation, which may change over time. GARFIELD is currently the largest ongoing international academic registry in patients with non-valvular AF; it represents a novel approach to outcomes research through the recruitment of unselected patients in five consecutive cohorts. In addition, through random selection of nationally representative sites, consecutive patient enrolment, and inclusion of patients perceived by their physicians to be at risk of stroke, the population will reflect those treated in everyday clinical practice. The contemporary data reported here provide a benchmark against which subsequent data, incorporating new therapies for AF, can be compared.

### Conclusions

These data highlight a substantial gap between evidence-based risk stratification, management recommendations, and their application in everyday clinical practice. The long-term implications of these management decisions will become apparent in the follow-up data at 1 and 2 years. With the introduction of new anticoagulants for AF, GARFIELD will describe how management strategies, patient outcomes, and use of healthcare resources evolve over time on a global level and in participating countries.

## Supporting Information

Ethics List S1 **Ethics committee information.**
(XLS)

Figure S1 **Year of diagnosis of AF in retrospective (part prospective) and prospective patients.**
(TIF)

Figure S2 **Distribution of (A) CHADS_2_ and (B) CHA_2_DS_2_-VASc Scores in the GARFIELD Registry.**
(TIF)

Table S1 **Patient baseline characteristics: retrospective (part prospective) validation group and prospective group.**
(DOC)

Text S1 **GARFIELD Registry Investigators.**
(DOC)
