# Supplementary figures and images for: Risk Profiles and Antithrombotic Treatment of Patients Newly Diagnosed with Atrial Fibrillation at Risk of Stroke: Perspectives from the International, Observational, Prospective GARFIELD Registry
Source: PLoS One. 2013 May 21;8(5):e63479. doi: 10.1371/journal.pone.0063479 (PMC3660389; doi:10.1371/journal.pone.0063479)

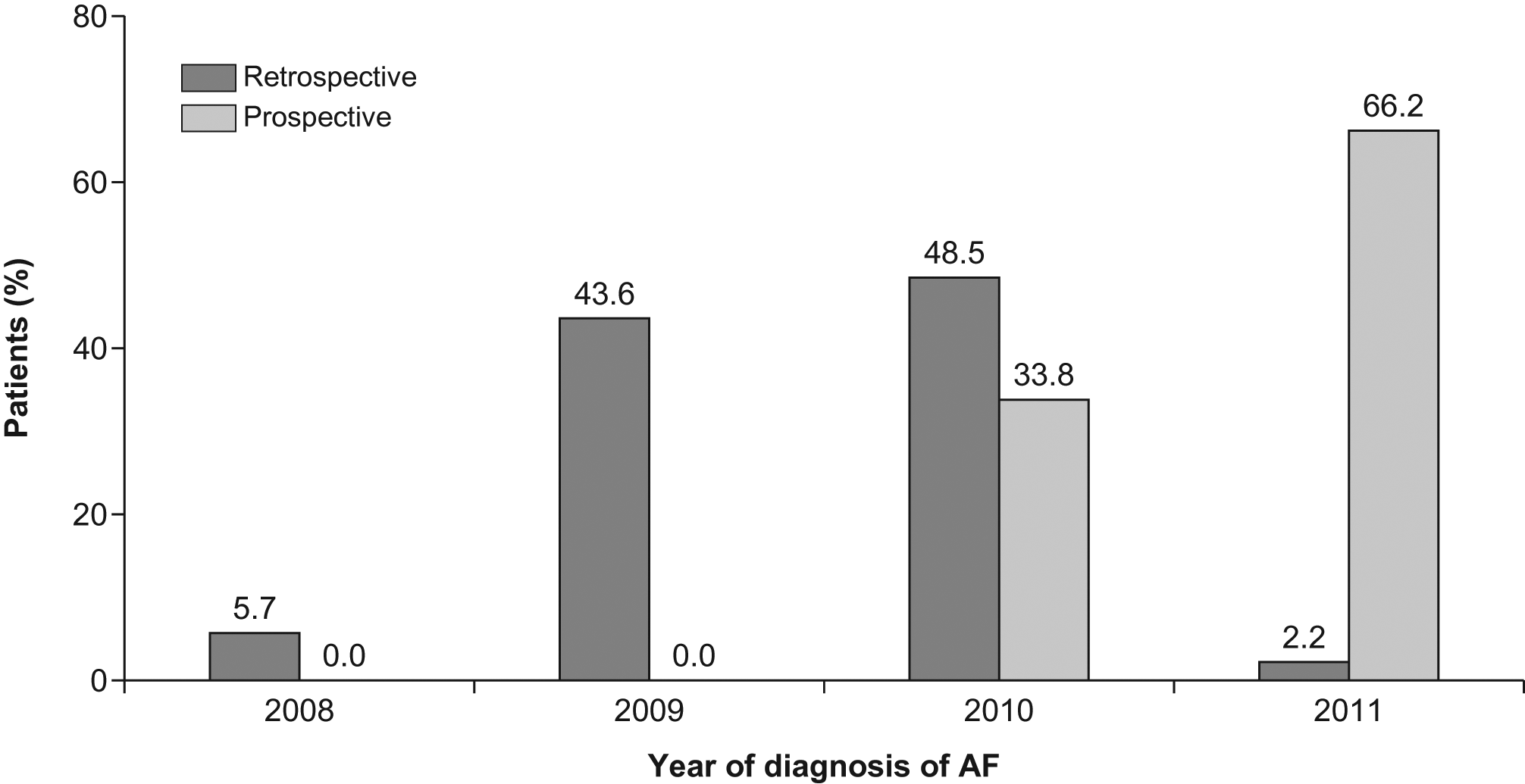

Supplement: Figure S1 — Year of diagnosis of AF in retrospective (part prospective) and prospective patients. (TIF) [file pone.0063479.s002.tif]

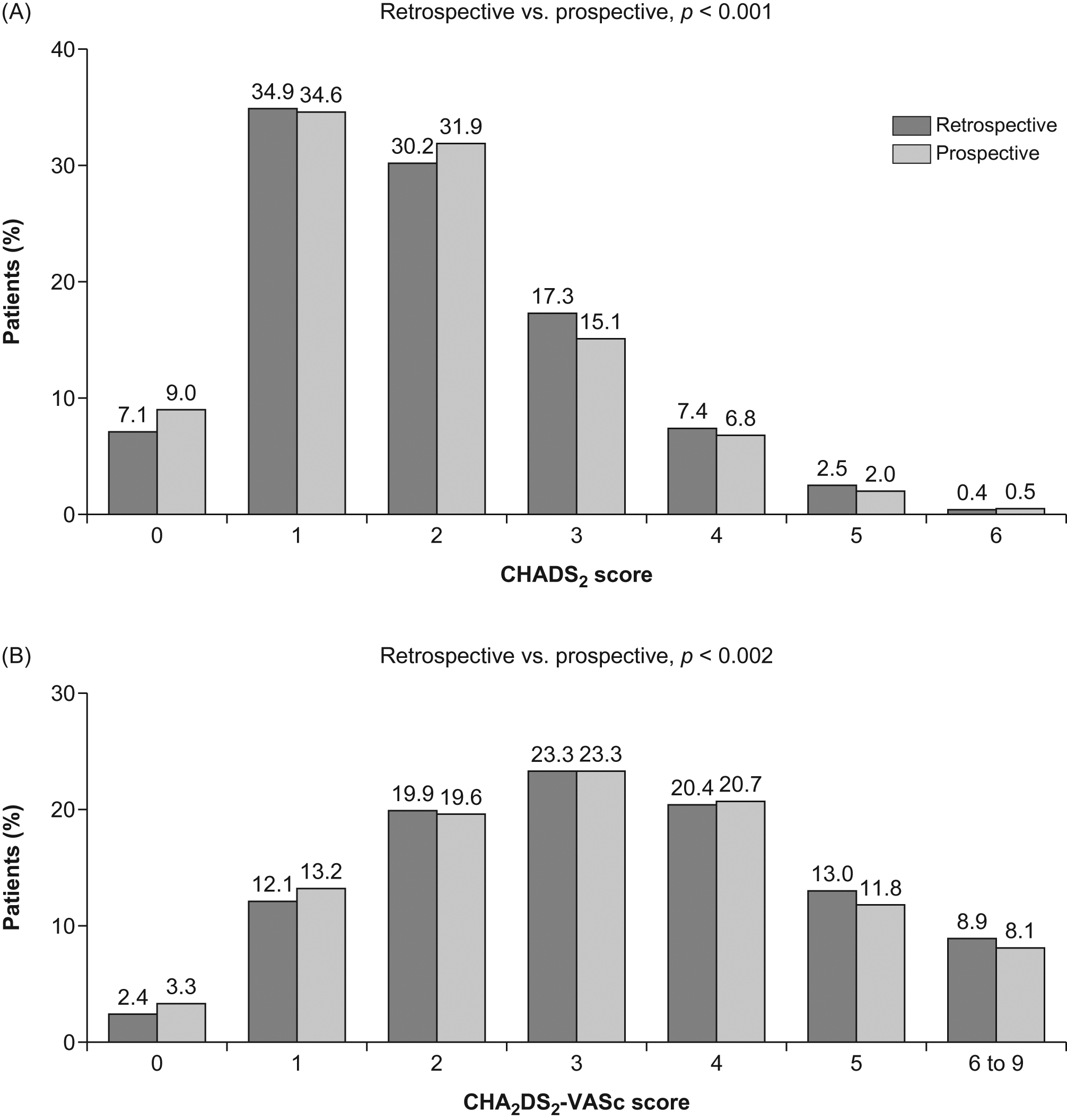

Supplement: Figure S2 — Distribution of (A) CHADS2 and (B) CHA2DS2-VASc Scores in the GARFIELD Registry. (TIF) [file pone.0063479.s003.tif]
