# Supplementary material for: Risk Profiles and Antithrombotic Treatment of Patients Newly Diagnosed with Atrial Fibrillation at Risk of Stroke: Perspectives from the International, Observational, Prospective GARFIELD Registry
Source: PLoS One. 2013 May 21;8(5):e63479. doi: 10.1371/journal.pone.0063479 (PMC3660389; doi:10.1371/journal.pone.0063479)
Supplement: Table S1 — Patient baseline characteristics: retrospective (part prospective) validation group and prospective group. (DOC) [file pone.0063479.s004.doc]

**Table S1. Baseline characteristics of the study population: retrospective and prospective cohorts.**

| **Variable** | **Retrospective cohort**  **(n = 5,089)** | **Prospective cohort**  **(n = 5,525)** | ***p* value**^a^ |
| --- | --- | --- | --- |
| Age, mean (SD), years | 70.6±10.8 | 69.8±11.5 | <0.001 |
| Age group, *n* (%) |  |  |  |
| >65 years | 3,581 (70.4) | 3,793 (68.7) | 0.055 |
| ≥75 years | 2,010 (39.5) | 2,081 (37.7) | 0.053 |
| 65–74 years | 1,702 (33.4) | 1,838 (33.3) | 0.85 |
| Women, *n* (%) | 2,168 (42.6) | 2,412 (43.7) | 0.27 |
| BMI,^b^ mean (SD), kg/m^2^ | 27.7±5.1 | 27.3±5.5 | <0.001 |
| Smoking status (current/previous),^c^ *n* (%) | 1,699 (35.9) | 1,805 (34.7) | 0.21 |
| Pulse,^d^ mean (SD), bpm | 84.5±23.9 | 88.5±25.8 | <0.001 |
| Medical history, *n* (%) |  |  |  |
| Congestive heart failure^e^ | 1,075 (21.1) | 1,154 (20.9) | 0.76 |
| Hypercholesterolemia^e^ | 2,127 (41.8) | 2,032 (36.8) | <0.001 |
| Hypertension^e^ | 4,002 (78.7) | 4,247 (76.9) | 0.025 |
| Acute coronary syndrome | 501 (9.8) | 559 (10.1) | 0.64 |
| Coronary artery disease^e^ | 969 (19.1) | 1,066 (19.3) | 0.75 |
| Family history of cardiac disease^a,f,g^ | 973 (19.1) | 967 (17.5) | 0.020 |
| Diabetes mellitus^g^ | 1,106 (21.8) | 1,224 (22.2) | 0.61 |
| Stroke^e^ history | 551 (10.8) | 475 (8.6) | <0.001 |
| Stroke or TIA^e^ history | 798 (15.7) | 730 (13.2) | <0.001 |
| LVEF <40%^b,h^ | 280 (9.9) | 306 (9.2) | 0.39 |
| Chronic renal disease^c,i^ |  |  |  |
| Mild renal dysfunction (GFR 60–89 mL/min) | 709 (19.4) | 793 (19.8) | 0.71 |
| Moderate renal dysfunction (GFR 30–59 mL/min) | 451 (12.4) | 420 (10.5) | 0.009 |
| Severe renal dysfunction or renal failure (GFR <30 mL/min) | 79 (2.2) | 75 (1.9) | 0.36 |
| Cirrhosis^e^ | 16 (0.3) | 39 (0.7) | 0.005 |
| Peripheral vascular/artery disease^e^ | 361 (7.1) | 382 (6.9) | 0.71 |
| Carotid occlusive disease^e^ | 177 (3.5) | 191 (3.5) | 0.95 |
| Other thromboembolism^d,e,j^ | 82 (1.6) | 68 (1.2) | 0.097 |
| Systemic embolism^e^ | 45 (0.9) | 35 (0.6) | 0.14 |
| History of bBleeding^e^ | 196 (3.9) | 172 (3.1) | 0.037 |
| Heavy alcohol consumption^e,k^ | 85 (1.9) | 130 (2.6) | 0.020 |
| PE or DVT^e^ history | 142 (2.8) | 162 (2.9) | 0.66 |
| Risk score, mean ± SD |  |  |  |
| CHADS_2_ | 2.0 (1.2) | 1.9 (1.2) | 0.001 |
| CHA_2_DS_2_-VASc | 3.3 (1.6) | 3.2 (1.6) | 0.006 |
| HAS-BLED | 1.1 (0.8) | 1.1 (0.8) | 0.056 |

Abbreviations: BMI, body mass index; DVT, deep vein thrombosis; GFR, glomerular filtration rate; LVEF, Left ventricular ejection fraction; PE, pulmonary embolism; SD, standard deviation; TIA, transient ischemic attack.

^a^Retrospective vs. prospective cohorts. By Chi-squared test for categorical variables, or unpaired t-test for continuous variables.

^b^Data not available for 933 and 678 patients in the retrospective and prospective cohorts, respectively.

^c^Data not available for 352 and 319 patients in the retrospective and prospective cohorts, respectively.

^d^Data not available for 840 and 532 patients in the retrospective and prospective cohorts, respectively.

^e^Data not available for 4 and 2 patients in the retrospective and prospective cohorts, respectively.

^f^First-degree relative with premature cardiac history (age <55 years [male], <65 years [female]).

^g^Data not available for 5 and 2 patients in the retrospective and prospective cohorts, respectively.

^h^Data not available for 2,247 and 2,201 patients in the retrospective and prospective cohorts, respectively.

^i^Data not available for 1,440 and 1,514 patients in the retrospective and prospective cohorts, respectively.

^j^For example, central venous thrombosis, retinal occlusion.

^k^Investigator defined; data not available for 556 and 492 patients in the retrospective and prospective cohorts, respectively.
