# Supplementary material for: Risk Profiles and Antithrombotic Treatment of Patients Newly Diagnosed with Atrial Fibrillation at Risk of Stroke: Perspectives from the International, Observational, Prospective GARFIELD Registry
Source: PLoS One. 2013 May 21;8(5):e63479. doi: 10.1371/journal.pone.0063479 (PMC3660389; doi:10.1371/journal.pone.0063479)
Supplement: Text S1 — GARFIELD Registry Investigators. (DOC) [file pone.0063479.s005.doc]

**Text S1. GARFIELD Registry Investigators**

**Global Steering Committee:** Professor the Lord Ajay K Kakkar (UK) (Chair), Jean-Pierre Bassand (France), A John Camm (UK), David A Fitzmaurice (UK), Samuel Z Goldhaber (USA), Shinya Goto (Japan), Sylvia Haas (Germany), Werner Hacke (Germany) Gregory YH Lip (UK), Lorenzo G Mantovani (Italy), Frank Misselwitz (Germany), Alexander GG Turpie (Canada), Martin van Eickels (Germany), Freek WA Verheugt (the Netherlands).

**Publications Committee:** Professor the Lord Ajay K Kakkar (UK) (Chair), Jean-Pierre Bassand (France), David A Fitzmaurice (UK), Samuel Z Goldhaber (USA), Shinya Goto (Japan), Sylvia Haas (Germany), Werner Hacke (Germany) Gregory YH Lip (UK), Lorenzo G Mantovani (Italy), Iris Mueller (UK), Frank Misselwitz (Germany), Sophie Rushton-Smith (UK), Alexander GG Turpie (Canada), Martin van Eickels (Germany), Freek WA Verheugt (the Netherlands).

**GARFIELD National Coordinators (Cohort 1)**

Antonio Carlos Pereira Barretto (Brazil), Carlos Jerjes Sánchez Díaz (Mexico), Dan Atar (Norway), David Fitzmaurice (UK), Dayi Hu (China), Giancarlo Agnelli (Italy), Harald Darius (Germany), Harry Gibbs (Australia), Hugo Ten Cate (the Netherlands), Janina Stepinska (Poland), Jean Yves le Heuzey (France), Jørn Dalsgaard Nielsen (Denmark), Marianne Brodmann (Austria), Marten Rosenqvist (Sweden), Pekka Raatikainen (Finland), Seil Oh (Korea), Stuart J Connolly, Alex Spyropoulos, John Eikelboom (Canada), Xavier Viñolas (Spain), Yukihiro Koretsune (Japan).

**GARFIELD National Investigators (Cohort 1)**

**AUSTRALIA:** Harry Gibbs, Adam Blenkhorn, Bhuwan Singh, William Van Gaal, Walter Abhayaratna, Ronald Lehman, Philip Roberts-Thomson, Jens Kilian, David Coulshed, Andrei Catanchin, David Colquhoun, Hosen Kiat, David Ecclestone, John French, Bronte Ayres, Thanh Phan, Peter Blombery, David Crimmins, David O’Donnell, Alice Choi, Penny Astridge.

**AUSTRIA:** Marianne Brodmann, Kurt Lenz, Heinz Drexel, Johannes Fochterle, Claus Hagn, Andrea Podczeck-Schweighofer, Kurt Huber, Michael Winkler, Bruno Schneeweiß, Alfons Gegenhuber, Wilfried Lang, Sabine Eichinger-Hasenauer, Peter Kaserer, Josef Sykora, Heribert Rasch, Max Pichler, Erich Schaflinger.

**BRAZIL:** Antonio Carlos Pereira Barretto**,** Dario Sobral-Filho, Jefferson Jaber, Dikran Armaganijan, José Faria Neto, Andre Steffens, Weimar Barroso, João Souza Neto, Jose Ribeiro, Marcelo Teixeira, Paulo Rossi, Leonardo Pires, Daniel Moreira, Jose Jorge, Adalberto Menezes Lorga Filho, Luiz Carlos Bodanese, Marcelo Montera, Carlos Del Carlo, Thiago Da Rocha Rodrigues, Fernando Costa, Antônio Lopes, Gilson Araújo, Euler Manenti, Jose Saraiva, João Braga, Alexandre Negri, Luiz Souto, Carlos Moncada, Dálton Précoma, Fernando Roquette, Gilmar Reis, Roberto Ramos, Estêvão Lanna Figueiredo, Roberto Betelho, Claudio Munhoz da Fontoura Tavares.

**CANADA:** Stuart J Connolly**,** Alex Spyropoulos, John Eikelboom Robert Luton, Milan Gupta, Shekhar Pandey, Stephen Cheung, Rolland Leader, Philippe Beaudry, Felix Ayala-Paredes, Jospeh Berlingieri, John Heath, Germain Poirier, Miranda Du Preez, Reginald Nadeau, George Dresser, Ripple Dhillon, Thomasz Hruczkowski, Bradley Schweitzer.

**CHINA**: Dayi Hu, KangNing Chen, Yu-Sheng Zhao, Huaiqin Zhang, JunZhu Chen, Shiping Cao, Daowen Wang, YueJin Yang, Weihua Li, YueHui Yin, Guizhou Tao, Ping Yang, Yingmin Chen, ShengHu He, Yong Wang, GuoSheng Fu, Xin Li, Tongguo Wu, Xiaoshu Cheng, Xiaowei Yan, Ruiping Zhao, Moshui Chen, Longgen Xiong, Ping Chen, Yang Jiao, Ying Guo, Li Xue, Fengzhi Wang, Hui Li.

**DENMARK:** Jørn Dalsgaard Nielsen, Henrik Nielsen, Steen Elkjær Husted, Ulrik Hintze, Søren Lind Rasmussen, Arno Bo Bremmelgaard, John Markenvard, Jan Boerger, Joergen Solgaard, Peter Simonsen, Thomas Loekkegaard, Michael Bruun, Jakob Mertz, Helena Domínguez.

**FINLAND:** Pekka Raatikainen and the Tampere GARFIELD investigator group.

**FRANCE:** Jean Yves le Heuzey, Michel Galinier, Yannick Gottwalles, Frank Paganelli, Philippe Loiselet, Jean-Joseph Muller, Bassel Koujan, Andre Marquand, Sylvain Destrac, Olivier Piot, Nicolas Delarche, Jean-Pierre Cebron, Serge Boveda, Maxime Guenoun, Dominique Guedj Meynier, Daniel Galley, Joel Ohayon, Serge Assouline, Mathieu Zuber, Pierre Amarenco, Emmanuel Ellie, James Kadouch, Pierre-Yves Fournier, Jean Pierre Huberman, Nestor Lemaire, Gilles Rodier, Loic Milandre, Xavier Vandamme, Igor Sibon, Jean Philippe Neau, Marie-Helene Mahagne, Antoine Mielot, Marc Bonnefoy, Jean-Baptiste Churet, Vincent Navarre, Frédéric Sellem, Gilles Monniot, Jean-Paul Boyes, Bernard Doucet, Michel Martelet, Désiré Obadia, Bernard Crousillat, Joseph Mouallem, Etienne Bearez, Pierre Nazeyrollas, Jean Philippe Brugnaux, Alain Fedorowsky.

**GERMANY:** Harald Darius, Georg Königer, Jan Purr, Uwe Gerbaulet, Thomas Kellner, Andreas Kopf, Thomas Schäfer, Hans Dieter Zauzig, Peter Riegel, Hartmut Hohensee, Enno Eißfeller, Wolfgang Eder, Günter Rehling, Dirk Glatzel, Stefan Zutz, Gerd-Ulrich Heinz, Holger Menke, Andreas Pustelnik, Petra Sandow, Norbert Ludwig, Henning Wiswedel, Wolfgang Wildenauer, Christoph Axthelm, Toralf Schwarz, Adyeri Babyesiza, Gerhard Stuchlik, Hans-Hermann Zimny, Maximilian Kropp, Friedhelm Kahl, Andreas Caspar, Sabine Omankowsky, Torsten Läßig, Hermann-Josef Hartmann, Günter Lehmann, Hans-Walter Bindig, Gunter Hergdt, Dietrich Reimer, Joachim Hauk, Wilfried Dorsch, Janna Dshabrailov, Holger Michel, Karl-Albrecht Rapp, Reinhold Vormann, Peter Mayer, Uwe Horstmeier, Volker Eissing, Karl-Heinz Hey, Heinz Leuchtgens, Volker Lilienweiß, Knut Kolitsch, Christian Schubert, Peter Hermann Lauer, Thomas Buchner, Gunter Brauer, Susanne Kamin, Karsten Müller, Muwafeg Abdel-Qader, Sylvia Baumbach, Hans-Holger Ebert, Carsten Schwencke, Sebastian Schellong, Peter Bernhardt, Laszlo Karolyi, Britta Sievers, Wilhelm Haverkamp, Peter Salbach , Jens-Uwe Röhnisch.

**ITALY:** Giancarlo Agnelli, Eros Tiraferri, Rita Santoro, Sophie Testa, Giovanni Di Minno, Marco Moia, Teresa Caimi, Giuliana Martini, Maria Tessitori,, Roberto Cappelli, Daniela Poli, Roberto Quintavalla, FrancescoMelone, Franco Cosmi, Attilia Pizzini, Gavino Piseddu, Raffaele Fanelli, Caterina Latella, Roberto Santi, Leonardo Pancaldi, Raimondo De Cristofaro, Gualtiero Palareti Angelo De Blasio, Jorge Salerno Uriarte, Franca Minetti, Enrico Maria Pogliani, Laura Maria Lonati, Michele Accogli, Nino Ciampani, Simona Malengo, Mauro Feola, Arturo Raisaro, Luciano Fattore, Piero Grilli, Fabrizio Germini, Maria Settimi, Massimo Alunni, Giuliana Duranti, Luca Tedeschi, Gregorio Baglioni, Giuseppe Avanzino, Mario Berardi, Valerio Pannacci, Angelo Giombolini, Sergio Nicoli, Tiziano Scarponi, Bartolomeo Allasia, Paulo Ricciarini, Roberto Nasorri, Antonio Argena, Pierdomenico Bossolasco, Paolo Ronchini, Alessandro Filippi, Filippo Tradati, Claudio Bulla, Luigi Donzelli, Luciano Foppa, Maria Luisa Bottarelli, Antonino Tomasello, Andrea Mauric.

**JAPAN:** Yukihiro Koretsune, Seisyu Kanamori, Kenichi Yamamoto, Kouichiro Kumagai, Yousuke Katsuda, Kenji Sadamatsu, Humitoshi Toyota, Yuji Mizuno, Ikuo Misumi, Hiroo Noguchi, Shinichi Ando, Tetsuro Suetsugu, Masahiro Minamoto, Hiroyuki Oda, Kouichi Shiraishi, Susumu Adachi, Kei Chiba, Hiroaki Norita, Makoto Tsuruta, Takeshi Koyanagi, Kunihiko Yamamoto, Hiroshi Ando, Takayuki Higashi, Kazuyoshi Okada, Shiro Azakami, Shinichiro Komaki, Koushi Kumeda, Takashi Murayama, Jyun Matsumura, Yurika Oba, Ryuji Sonoda, Kazuo Goto, Kotaro Minoda, Yoshikuni Haraguchi, Hisakazu Suefuji, Hiroo Miyagi, Hitoshi Kato, Tsugihiro Nakamura, Tadashi Nakamura, Hidekazu Nandate, Ryuji Zaitsu, Yoshihisa Fujiura, Akira Yoshimura, Hiroyuki Numata, Jun Ogawa, Hiroshi Tatematsu, Yasuyuki Kamogawa, Kinshiro Murakami, Yutaka Wakasa, Masanori Yamasawa, Hiromitsu Maekawa, Sumihisa Abe, Hajime Kihara, Satoru Tsunoda, Katsumi Saito, Tetsuro Fudo.

**KOREA:** Seil Oh, Hui-Nam Pak, Jin-Bae Kim, Jeong Hoon Kim, Sung-Won Jang, Dae Hyeok Kim, Jun Kim, Dong Ryeol Ryu, Sang Won Park, Daekyeong Kim, Dong-Ju Choi, Yong Seog Oh, Myeong-Chan Cho, Sang-Hyun Kim, Hui Kyung Jeon, Dong-Gu Shin, Jin Sik Park, Hoon Ki Park, Sang-Jin Han, Jung Hoon Sung, Jeong Gwan Cho, Gi-Byoung Nam, Young Keun On, Hong Euy Lim, Jae Jin Kwak, Tae-Joon Cha, Taek Jong Hong, Seong Hoon Park, Junghan Yoon, Nam-Ho Kim, Kee-Sik Kim, Byung Chun Jung, Gyo-Seung Hwang, Chong-Jin Kim, Jae Seok Park.

**MEXICO:** Carlos Jerjes Sánchez Díaz, Jesús Illescas Diaz, Raúl Cantú, María Guadalupe Ramos Zavala, Ricardo Cabrera Jardines, Nilda Espinola Zavaleta, Sergio Villarreal Umaña, Enrique López Rosas, Guillermo Llamas Esperón, Gerardo Pozas, Ernesto Cardona Muñoz, Norberto Matadamas Hernández, Adolfo Leyva Rendón, Norberto García Hernández, Manuel Odín de los Ríos Ibarra, Luis Virgen Carrillo, David López Villezca, Carlos Hernández Herrera, Juan-José López-Prieto, Rodolfo Gaona Rodríguez, Efraín Villeda Espinosa, David Flores Martínez, Jose Velasco Barcena, Rúben Yong, Ignacio Rodríguez Briones, José-Luís Leiva-Pons, Humberto Álvarez López, Rafael Olvera-Ruiz, Carlos Díaz de la Vega, Carlos Cantú Brito, Eduardo Chuquiure Valenzuela.

**THE NETHERLANDS:** Hugo Ten Cate, Jan Ruiter, Huber Klomps, Maarten Bongaerts, M Mattie GC Pieterse, Coen Guldener, Jean-Paul R Herrman, Gerben Lochorn.

**NORWAY:** Dan Atar, Eivind Berge, Per Anton Sirnes, Erik Gjertsen, Torstein Hole, Knut Erga, Arne Hallaråker, Gunnar Skjelvan, Anders Østrem, Beraki Ghezai, Arne Svilaas, Peter Christersson, Torbjørn Øien, Svein Høegh Henrichsen.

**POLAND:** Janina Stepinska, Romuald Korzeniak, Andrzej Gieroba, Malgorzata Biedrzycka, Michal Ogorek, Beata Wozakowska-Kaplon, Krystyna Loboz-Grudzien, Jaroslaw Kosior, Wieslaw Supinski, Jerzy Kuzniar, Roman Zaluska, Jaroslaw Hiczkiewicz, Lucyna Swiatkowska-Byczynska, Lech Kucharski, Marcin Gruchala, Piotr Minc, Maciej Olszewski, Grzegorz Kania, Malgorzata Krzciuk, Zbigniew Lajkowski, Bozenna Ostrowska-Pomian, Jerzy Lewczuk, Elzbieta Zinka, Agnieszka Karczmarczyk, Malgorzata Chmielnicka-Pruszczynska, Maria Trusz-Gluza, Grzegorz Opolski, Marek Bronisz, Marcin Ogorek, Grazyna Glanowska, Piotr Ruszkowski, Krystyna Jaworska, Ryszard Sciborski, Boguslaw Okopien, Piotr Kukla.

**SPAIN:** Xavier Viñolas, Pere Alvarez, María López Fernández, Luis Tercedor, Salvador Tranche, Pere Torán, Emilio Márquez Contreras, Jordi Isart Rafecas, Juan Motero-Carrasco, Pablo García Pavía, Casimiro Gómez Pajuelo, Concepcion Moro, Luis Iglesias Alonso, Ángel Grande, Jordi Mercé, Jose Ramon Gonzalez Juanatey, Gonzalo Barón, Ines Monte Collado, Herminia Palacín Piquero, Carles Brotons Cuixart, Maria Rodríguez Morató, Joan Bayo I Llibre, Cecilia Corros Vicente, Manuel Vida, Francisco Epelde Gonzalo, Carlos Alexandre Almeida Fernández, Nuria Del Val Plana, Enrique Escrivá Montserrat, Juan Montero Alía, María Barreda González, María Moleiro Oliva, José Iglesias Sanmartín, Mercedes Jiménez González, María Rodriguez Álvarez, Juan Herreros Melenchon.

**SWEDEN:** Marten Rosenqvist, Alexander Wirdby, Jan Linden, Kerstin Henriksson, Micael Elmersson, Arnor Egilsson, Ulf Börjesson, Gunnar Svärd, Bo Liu, Anders Lindh, Lars-Bertil Olsson, Mikael Gustavsson, Lars Andersson, Lars Benson, Claes Bothin, Ali Hajimirsadeghi, Khyam Kadir, Marianne Ericsson, Åke Ohlsson, Hakan Lindvall, Peter Svensson, Katarina Thorne, Hans Handel, Pyotr Platonov, Björn Eriksson, Ingar Timberg, Kerstin Romberg, Milita Crisby.

**UNITED KINGDOM:** David Fitzmaurice at the University of Birmingham and the UK Clinical Research Network (Primary care). Naresh Chaunan, Daryl Goodwin, Philip Saunders, Richard Evans, Philip Saunders, Jante Leese, Prem Jhittay, Anew Ross, Manjit Kainth, Gill Pickavance, Joanna McDonnell, Andrea Williams, Trevor Gooding, Helga Wagner, Shoeb Suryani, Arun Singal, Subhasri Sircar, Roman Bilas, Peter Hutchinson, Anne Wakeman, Michael Stokes, Neil Paul, Michael Aziz, Cobarsanellore Ramesh, Peter Wilson, Simon Franklin, Sue Fairhead, Julian Thompson, Vivien Joseph, Gary Taylor, Dawn Tragen, David Seamark, Carolyn Paul, Mark Richardson, Angus Jefferies, Helen Sharp, Hywel Jones, Claire Giles, Michael Page, Olaleye Oginni, Jehad Aldegather, Simon Wetherwell, William Lumb, Phil Evans, Frances Scouller, Neil Macey, Yvette Stipp, Richard West, StephenThurston, Paul Wadeson, John Matthews, Preeti Pandya, Andrew Gallagher, Tammy Railton, Bijoy Sinha, David Russell, Jason Davies, Ainsworth, Claire Jones, Phil Weeks , Jane Eden, David Kernick.
